# Quantitative bone SPECT/CT: high specificity for identification of prostate cancer bone metastases

**DOI:** 10.1186/s12891-019-3001-6

**Published:** 2019-12-26

**Authors:** Flavian Tabotta, Mario Jreige, Niklaus Schaefer, Fabio Becce, John O. Prior, Marie Nicod Lalonde

**Affiliations:** Lausanne University Hospital and University of Lausanne, Rue du Bugnon 46, 1011 Lausanne, Switzerland

**Keywords:** SPECT/CT, SUV, Bone metastases, Prostate cancer, Bone scintigraphy, Spinal osteoarthritis, ^99m^Tc-DPD, xSPECT, Quantitative SPECT, Prostate cancer metastases

## Abstract

**Purpose:**

Bone scintigraphy with ^99m^Tc-labeled diphosphonates can identify prostate cancer bone metastases with high sensitivity, but relatively low specificity, because benign conditions such as osteoarthritis can also trigger osteoblastic reactions. We aimed to investigate the diagnostic performance of ^99m^Tc-2,3-dicarboxy propane-1,1-diphosphonate (^99m^Tc-DPD) uptake quantification by single-photon emission computed tomography coupled with computed tomography (SPECT/CT) for distinguishing prostate cancer bone metastases from spinal and pelvic osteoarthritic lesions.

**Methods:**

We retrospectively assessed 26 bone scans from 26 patients with known prostate cancer bone metastases and 13 control patients with benign spinal and pelvic osteoarthritic changes without known neoplastic disease. Quantitative SPECT/CT (xSPECT, Siemens Symbia Intevo, Erlangen, Germany) was performed and standardized uptake values (SUVs) were quantified with measurements of SUV_max_ and SUV_mean_ (g/mL) in all bone metastases for the prostate cancer group and in spinal and pelvic osteoarthritic changes for the control group. We used receiver operating characteristics (ROC) curves to determine the optimum SUV_max_ cutoff value to distinguish between bone metastases and benign spinal and pelvic lesions.

**Results:**

In total, 264 prostate cancer bone metastases were analyzed, showing a mean SUV_max_ and SUV_mean_ of 34.6 ± 24.6 and 20.8 ± 14.7 g/mL, respectively. In 24 spinal and pelvic osteoarthritic lesions, mean SUV_max_ and SUV_mean_ were 14.2 ± 3.8 and 8.9 ± 2.2 g/mL, respectively. SUV_max_ and SUV_mean_ were both significantly different between the bone metastases and osteoarthritic groups (*p* ≤ 0.0001). Using a SUV_max_ cutoff of 19.5 g/mL for prostate cancer bone metastases in the spine and pelvis, sensitivity, specificity, positive and negative predictive values were 87, 92, 99 and 49%, respectively.

**Conclusion:**

This study showed significant differences in quantitative ^99m^Tc-DPD uptake on bone SPECT/CT between prostate cancer bone metastases and spinal and pelvic osteoarthritic changes, with higher SUV_max_ and SUV_mean_ in metastases. Using a SUV_max_ cutoff of 19.5 g/mL, high specificity and positive predictive value for metastases identification in the spine and pelvis were found, thus increasing accuracy of bone scintigraphy.

## Introduction

In developed countries, prostate cancer is the most frequently diagnosed cancer among men and the fifth leading cause of cancer death [1]. Bone is the main site of distant metastases [2], with a high yet underreported prevalence [3]. Standard initial local treatment options include watchful waiting, radiation therapy and radical prostatectomy. Recurrence is detected by serum elevation of prostate specific antigen (PSA). Accurate initial staging and restaging, namely the detection of bone metastases, is essential for choosing the most appropriate treatment for the patient.

^99m^Tc-diphosphonates bone scintigraphy is the most widely available imaging modality worldwide to detect bone metastases in patients with prostate cancer. Bone scintigraphy uses DPD-labelled with ^99m^Tc, which accumulate in remodeling bone by incorporation into the crystalline structure of calcium hydroxyapatite [3]. ^99m^Tc-DPD bone uptake depends on bone osteoblastic activity, vascularization and environmental factors. Bone metastases of prostate cancer trigger an important osteoblastic reaction and substantially accumulate ^99m^Tc-DPD. Planar bone scintigraphy has a high sensitivity but a relatively low specificity for characterizing bone metastases in prostate cancer patients. Indeed, benign conditions, such as degenerative joint and disk diseases, also trigger an increase in bone turnover and radiotracer accumulation. The 3D data acquired during bone scintigraphy, named single-photon emission computed tomography (SPECT), can be coupled with computed tomography (CT), a morphological imaging modality. It is well known that combined SPECT/CT increases the specificity of bone scintigraphy as the sites of increased ^99m^Tc-DPD uptake can be correlated to morphological changes on CT images [4–6].

Recently, technological advances have allowed ^99m^Tc-DPD uptake quantification (xSPECT/CT, Siemens Symbia Intevo). xSPECT has an accurate activity recovery within 10% of the expected value for objects > 10 mL, which is similar to PET/CT [7]. The aim of this study was to investigate the diagnostic performance of ^99m^Tc-DPD uptake quantification for distinguishing bone metastases from benign spinal and pelvic osteoarthritic lesions in prostate cancer patients.

## Materials and methods

### Patient selection

We retrospectively analyzed 26 bone scans from 26 male patients (mean age 74 ± 10 years; range 55–92 years) with confirmed prostate cancer on biopsy or based on biological data and imaging follow-up, referred for evaluation of bone metastases between January 2016 and December 2018. The second group consisted of 13 male patients (70 ± 15 years; range 32–83 years) without any known neoplastic disease, referred for investigation of various benign musculoskeletal disorders. Patient data, including body mass index (BMI), administered ^99m^Tc-DPD activity, creatinine levels, PSA levels and time interval between radiotracer injection and image acquisition were retrieved.

### SPECT/CT image analysis

All patients underwent whole-body planar imaging with the low-energy high-resolution collimator with a scanning speed of 12 cm/min and quantitative xSPECT/CT (Siemens Symbia Intevo, Erlangen, Germany) on regions with high uptake on planar scintigraphy. The xSPECT was acquired in average at 3 h35 ± 54 min in the bone metastases group and 3 h50 ± 50 min in the osteoarthritic group, after intravenous injection of 10 MBq/kg of ^99m^Tc-DPD (this radiotracer has been in use in our center for over 2 decades, as we believe the bone/soft tissue ratio is better) with a mean patient dose of 777 ± 113 MBq and 733 ± 101 MBq, respectively. Images were acquired with 3 degrees rotation/step and 12 s/projection with a 256 × 256 matrix. Reduced dose CT was acquired with 130 kV and 25 reference mAs modulation (Siemens Care Dose, Symbia Intevo, Erlangen, Germany). Images were reconstructed to generate SPECT data allowing SUV_bw_ quantification on post-processed images and measurement of SUV_max_ and SUV_mean_ (g/mL) using xSPECT reconstruction algorithm.

**Fig. 1:**
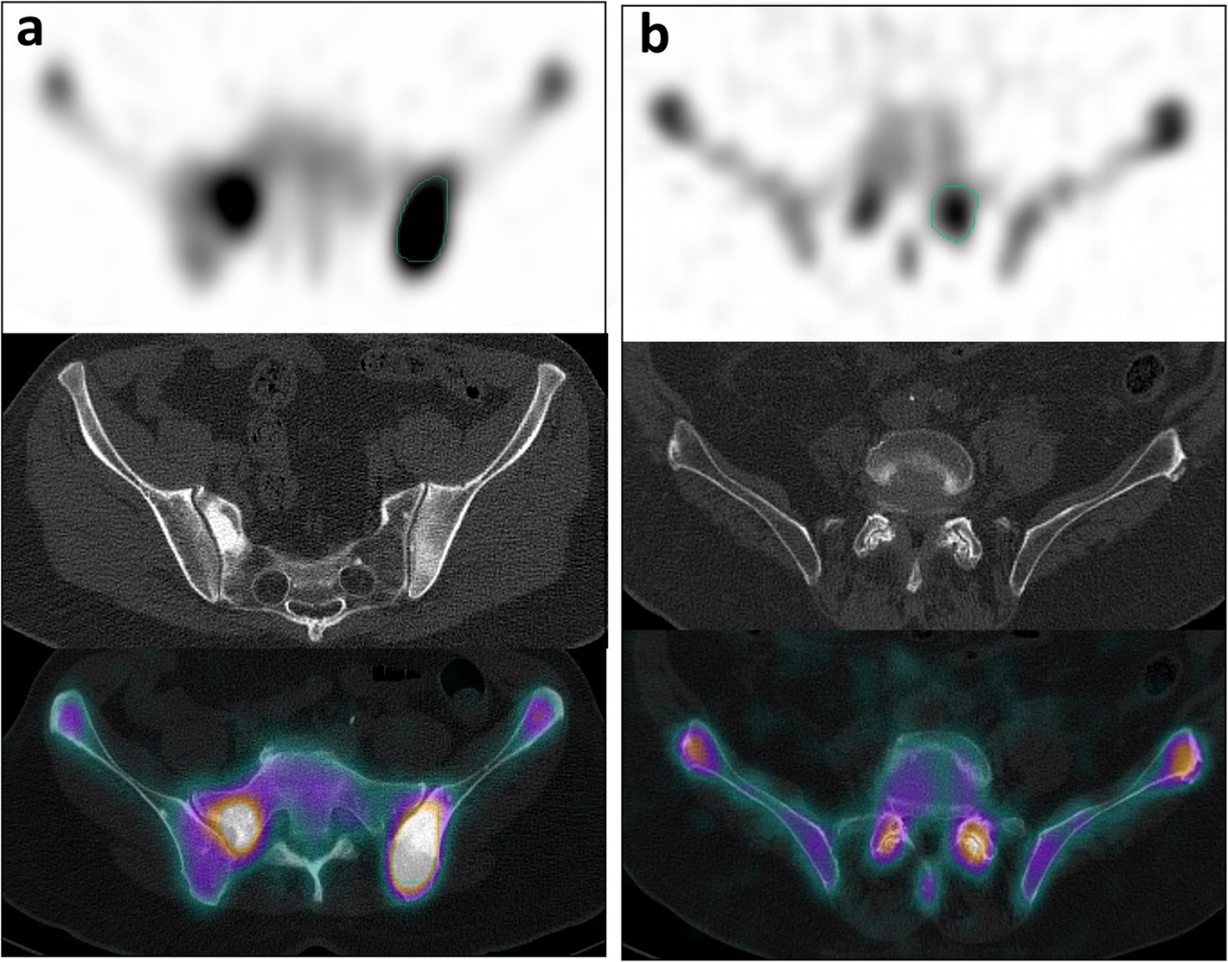
**a** Osteoblastic lesion of the pelvis in a 57-year-old male patient known for prostate cancer, showing a high SUV_max_ of 28 g/mL and SUV_mean_ of 17 g/mL. **b** Lumbar spine osteoarthritic changes of the L4-L5 facet joints in a 83-year-old male patient with hip pain, showing SUV_max_ of 15 and 13 g/mL and SUV_mean_ of 9.0 and 7.8 g/mL in the left and right facet joints, respectively

For each patient in the bone metastases group, SUV_max_ and SUV_mean_ of all prostate cancer bone metastases visible on SPECT and CT were measured (Fig. 1a). For each patient in the control group, SUV_max_ and SUV_mean_ were measured in the active degenerative changes of the lumbar spine and pelvis on SPECT/CT (Fig. 1b). For all patients, the SUV_mean_ of lumbar vertebrae was measured in a 4 to 5 cm^3^ region of interest (ROI), with no metastatic or osteoarthritic lesion visible on SPECT and CT.

### Statistical analysis

Statistical differences between bone metastases and osteoarthritic groups regarding age, BMI, creatinine levels, lumbar vertebrae SUV_max_ and SUV_mean_, time interval between radiotracer injection and image acquisition, were calculated using the Wilcoxon rank-sum test. Mean values, standard deviations (SD) of SUV_max_ and SUV_mean_ in both metastatic and osteoarthritic groups were calculated and statistical differences assessed by the Wilcoxon rank-sum test. Subgroup analysis taking into account the metastases location was performed in the bone metastases group. Differences in SUV_max_ and SUV_mean_ between subgroups were assessed using one-way ANOVA. We also used receiver operating characteristics (ROC) curves to determine the best-fit cutoff values of SUV_max_ between metastatic and osteoarthritic lesions, with computation of the respective sensitivity, specificity, positive and negative predictive values. All statistical analyses were performed using STATA (version 15.1; STATA Corp., College Station, Texas, USA). *P-*values less than 0.05 were considered as statistically significant.

## Results

There were no significant differences between the bone metastases and osteoarthritic groups regarding age, BMI, creatinine levels, time between radiotracer injection and xSPECT imaging and lumbar vertebrae SUV_max_ and SUV_mean_ (Table 1). The average lumbar vertebrae SUV_max_ and SUV_mean_ of all patients were 8.8 ± 2.3 and 6.9 ± 1.9 g/mL, respectively. The PSA level in the metastatic group was 206 ± 573 μg/L.

**Table 1:** Patient characteristics in the prostate cancer bone metastases and spinal and pelvic osteoarthritis groups

	Bone Metastases Group (*n* = 26)	Osteoarthritis Group (*n* = 13)	*P*-value
Age (years)	74 ± 10	70 ± 15	0.68
BMI (kg/m^2^)	25.6 ± 4.9	25 ± 3.2	0.87
Creatinine level (μmol/L)	92.3 ± 27.4	96.1 ± 33.3	0.66
Time interval between injection and xSPECT imaging (min)	215 ± 54	230 ± 50	0.32
SUV_mean_ lumbar vertebrae	6.7 ± 1.9	7.3 ± 1.9	0.26
SUV_max_ lumbar vertebrae	8.6 ± 2.4	9.2 ± 2.1	0.35

**Fig. 2:**
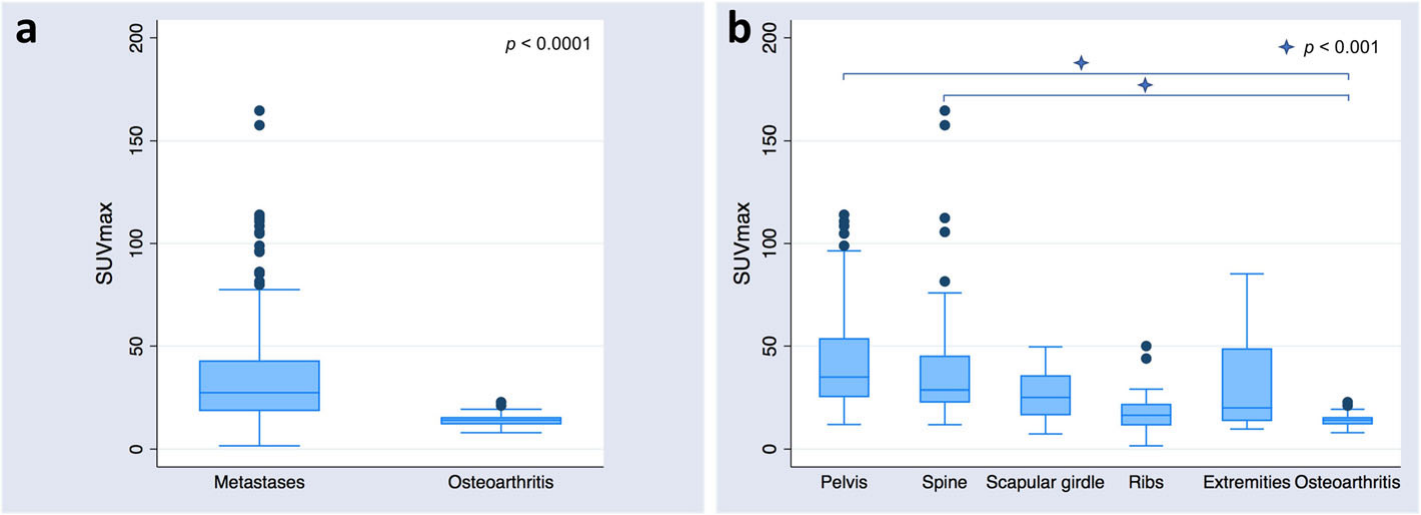
Box-and-whisker plots of SUV_max_ overall (**a**) and depending on metastases location (**b**)

A total number of 265 prostate cancer bone metastases (221 osteoblastic, 5 osteolytic, 35 mixed and 4 non-classified) were analyzed, showing a mean SUV_max_ and SUV_mean_ of 35 ± 25 and 21 ± 15 g/mL, respectively. In the osteoarthritic group, 24 active focal osteoarthritic changes (20 spinal and 4 pelvic) were analyzed and showed a mean SUV_max_ and SUV_mean_ of 14.2 ± 3.8 and 8.9 ± 2.2 g/mL, respectively. SUV_max_ and SUV_mean_ were both significantly different between bone metastatic and osteoarthritic lesions (*p* < 0.0001) (Fig. 2a).

In the bone metastases group, there were 87 lesions in the pelvis (SUV_max_ 44 ± 25 g/mL and SUV_mean_ 26 ± 15 g/mL), 84 lesions in the spine (SUV_max_ 39 ± 28 g/mL and SUV_mean_ 24 ± 17 g/mL), 28 lesions in the scapular girdle (SUV_max_ 27 ± 12 g/mL and SUV_mean_ 16 ± 7.2 g/mL), 54 lesions in the ribs (SUV_max_ 18 ± 8.9 g/mL and SUV_mean_ 10.6 ± 5.1 g/mL), and 12 lesions in the extremities (SUV_max_ 33 ± 26 g/mL and SUV_mean_ 20 ± 16 g/mL). SUV_max_ and SUV_mean_ of metastatic lesions in the spine and pelvis were significantly higher than in osteoarthritic lesions (*p* < 0.0001), whereas there was no significant difference between SUVs of osteoarthritic changes and metastatic lesions in the ribs, scapular girdle, and the extremities (*p* = 0.53, *p* = 1.0 and *p* = 0.3, respectively) (Fig. 2b).

**Fig. 3:**
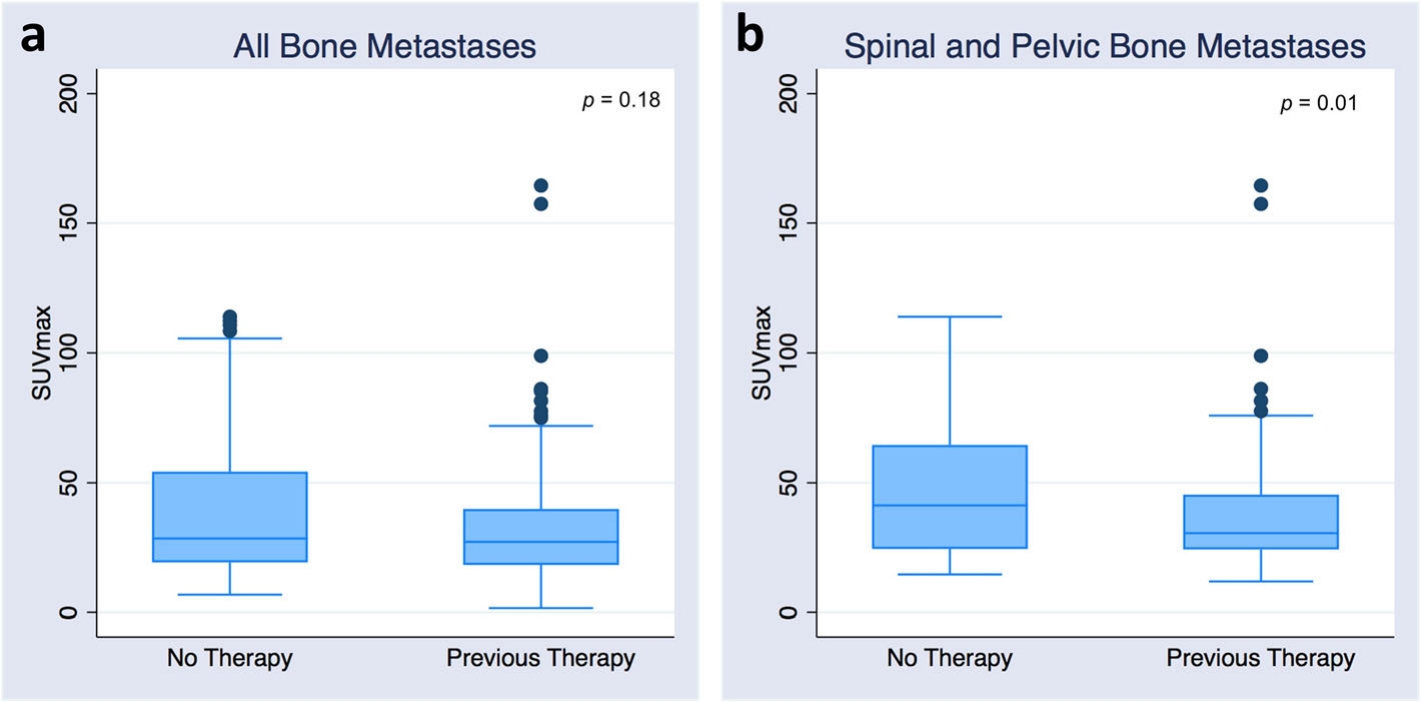
Box-and-whisker plots of SUV_max_ depending on presence or absence of previous systemic therapy in all bone metastases (**a**) and in spinal and pelvic bone metastases (**b**)

In the bone metastases group, 16 patients had previous therapy before relapse. All 16 patients had hormone therapy, one patient had Xofigo (^223^Radium dichloride) and immunotherapy prior to bone scan and another patient had chemotherapy. SUV_max_ and SUV_mean_ of the bone metastases were not significantly lower in patients having had previous systemic therapy compared to patients without previous treatment (SUV_max_ 32 ± 22 versus 40 ± 29 g/mL, *p* = 0.18, and SUV_mean_ 20 ± 14 versus 23 ± 17 g/mL, *p* = 0.3) (Fig. 3a). Interestingly, this difference became significant when only spinal and pelvic bone metastases were considered: SUV_max_ 37 ± 24 versus 50 ± 30 g/mL (*p* = 0.01) and SUV_mean_ 23 ± 14 versus 30 ± 18 g/mL (*p* = 0.03), respectively (Fig. 3b).

**Fig. 4:**
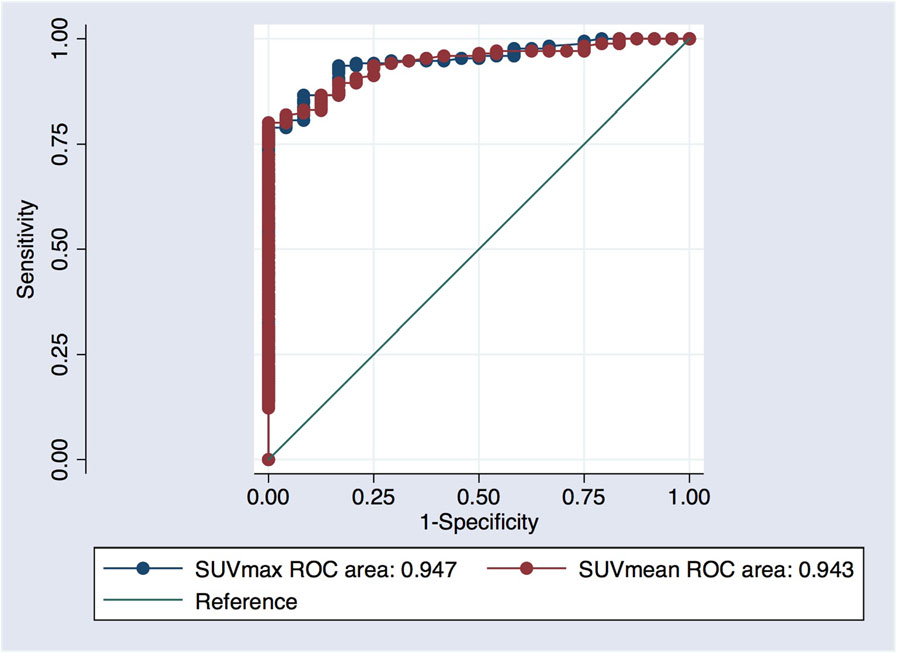
ROC curves of SUV_max_ and SUV_mean_ for spinal and pelvic prostate cancer bone metastases, with AUCs of 0.947 and 0.943, respectively

ROC curves showed that both SUV_max_ and SUV_mean_ had very good diagnostic accuracy for differentiating between spinal and pelvic bone metastases and osteoarthritic changes (AUC 0.947 and 0.943, respectively) (Fig. 4). The optimum cutoff value of SUV_max_ for defining spinal and pelvic prostate cancer bone metastases was 19.5 g/mL. Using this cutoff value, we found a sensitivity, specificity, positive and negative predictive values of 87% [95% CI: 81–91%], 92% [73–99%], 99% [95–100%] and 49% [34–64%], respectively. This cutoff value remained identical even in patients who had prior therapy, with a sensitivity, specificity, positive and negative predictive values of 86% [95% CI: 78–91%], 92% [73–99%], 98% [93–100%] and 56% [40–72%], respectively.

## Discussion

In this study, we showed that quantification in bone scintigraphy could help in distinguishing prostate cancer bone metastases from spinal and pelvic osteoarthritic changes, and therefore increase bone scan specificity. Using an optimum SUV_max_ cutoff of 19.5 g/mL for defining spinal and pelvic bone metastases on SPECT/CT, bone scan yielded a sensitivity, specificity, positive and negative predictive values of 87, 92, 99 and 49%, respectively.

To the best of our knowledge, only few studies previously reported on the quantification of ^99m^Tc-DPD uptake in bone metastases of prostate cancer patients. Beck et al. showed that the mean SUV_peak_ of metastatic lesions in breast and prostate cancer patients was 20.4 ± 20.8 g/mL [8]. This difference is at least partly due to the fact that SUV_peak_ is usually lower than SUV_max_. Moreover, patients included in their study were both prostate and breast cancer patients combined, with a majority of the latter. Since breast cancer metastases are both osteoblastic and osteolytic, their uptake is probably lower than that of prostate cancer metastases, which are mostly osteoblastic as in our patient population. SUVs were further higher in our study than those reported by Umeda et al., who found a lower threshold of 7 g/mL of SUV_max_, above which the tumor burden of metastatic prostate cancer patients was determined [9]. Kuji et al. reported a high accuracy of quantitative SPECT/CT for the diagnosis of bone metastases in 170 prostate cancer patients [10]. They used a different reconstruction algorithm based on CT zonal mapping and included only the three hottest lesions explaining a slightly higher SUV_max_ and SUV_mean_ (SUV_max_ of 35 ± 25 g/mL in our study versus 41 ± 34 g/mL in the study by Kuji et al., and a mean SUV_mean_ of 21 ± 15 g/mL versus 24.6 ± 21.2 g/mL, respectively). We had similar quantitative results compared to Kuji et al. regarding radiotracer uptake by spinal osteoarthritic changes with a mean SUV_max_ of 14.2 ± 3.8 g/mL compared to 16.7 ± 6.7 g/mL, and a mean SUV_mean_ of 8.9 ± 2.2 g/mL compared to 9.5 ± 3.9 g/mL, respectively. Interestingly, SUVs were comparable although the radiotracers used for the bone scan were slightly different: ^99m^Tc-DPD versus ^99m^Tc-methylene diphosphonate (^99m^Tc-MDP) in the Kuji et al. study. Thus, SUVs seem not only comparable from one center to another but also comparable regardless of which type of diphosphonate is used. This is further reinforced by comparable lumbar vertebrae SUV_mean_ in our study compared to the vertebral SUV_mean_ in the study by Cachovan et al. (SUV_mean_ 6.9 ± 1.9 in our study versus 5.91 ± 1.54 in the Cachovan et al. study) [11].

In our study, SUV_max_ and SUV_mean_ of metastatic lesions varied depending on the lesion location, with lowest values in the ribs and scapular girdle, which is similar to the observations from Beck and al [8]. This is probably at least partly due to the size of the lesions, which tend to be smaller in the ribs and scapular girdle with partial volume effect, as described for PET [12]. Different vascularization and osteoblastic reaction in these different anatomical regions may also play a role. There was no significant difference in SUV_max_ and SUV_mean_ between metastatic lesions in the ribs, scapular girdle and extremities, and osteoarthritic lesions; hence, no cutoff value could be obtained to distinguish between them. For lesions in the spine and pelvis, we found an optimum SUV_max_ cutoff of 19.5 g/mL with a very high sensitivity and positive predictive value. We believe this cutoff could be of added diagnostic value enabling physicians to decide with a high accuracy that a focal ^99m^Tc-DPD uptake in the spine or pelvis above 19.5 g/mL is most likely to be metastatic in a patient with known prostate cancer.

In this study, SUV_max_ was significantly lower in spinal and pelvic bone metastases of patients relapsing after prior systemic therapy compared to patients with no previous systemic treatment. This is not surprising as previous treatment may induce sclerosis, reduced vascularization, or other tumor environment changes, which can all influence ^99m^Tc-DPD uptake. Understanding the mechanism of reduced uptake in these lesions may help better understand the bone tumor microenvironment of prostate metastases [13]. Interestingly, the SUV_max_ cutoff of 19.5 g/mL still had a high positive predictive value for spinal and pelvic bone metastases in patients having received prior treatment for bone metastases.

There are of course other nuclear medicine modalities available for the detection and characterization of bone metastases in prostate cancer patients. ^68^Ga-prostate specific membrane antigen (PSMA), ^18^F-choline and ^18^F-sodium fluoride (NaF) PET/CT all have very good accuracies for the diagnosis of prostate cancer bone metastases [14–17]. The most promising modality seems to be ^68^Ga-PSMA PET/CT [18, 19]. Nonetheless, in many countries, ^68^Ga-PSMA, ^18^F-Choline and ^18^F-NaF PET are not widely available for primary staging due to cost and reimbursement issues, as impact on patient management and cost-effective studies are not yet available [20]. A study comparing PSA cutoff value for ordering ^18^F-NaF PET or bone scintigraphy in patients with newly diagnosed prostate cancer showed no major difference between both modalities [20]. Furthermore, a recent study by Arvola et al. showed a strong correlation between SUVs from ^99m^Tc-HDP SPECT/CT and ^18^F-NaF PET/CT [21]. The authors concluded that SPECT is an applicable tool for clinical quantification of bone metabolism in osseous metastases in breast and prostate cancer patients.

Therefore, quantitative bone scintigraphy seems to increase bone SPECT/CT accuracy, allowing bone scintigraphy to remain competitive in the era of new multimodality imaging of bone metastases in prostate cancer patients. We believe that the SUV_max_ cutoff of 19.5 mg/L for spinal and pelvic lesions could further increase bone scan specificity.

The main limitations of our study are the small subject population and the lack of histological confirmation for all prostate cancer bone metastases. However, as authors reported that histological confirmation may be avoided in the case of typical morphological imaging findings and patterns of radiotracer uptake [22], lesions were thus diagnosed as metastatic on conventional SPECT/CT and were subsequently analyzed for tracer uptake quantification. In addition, since we proceeded with a lesion-based analysis, the relatively small number of patients did not allow correlating further the level of uptake with the different histological grades of prostate cancer.

## Conclusion

This study demonstrated significant differences in ^99m^Tc-DPD uptake on bone scan between prostate cancer bone metastases and spinal and pelvic osteoarthritic changes based on quantitative data analysis, with significantly higher SUV_max_ and SUV_mean_ in metastases. Using an optimum SUV_max_ cutoff of 19.5 g/mL for defining spinal and pelvic bone metastases on SPECT/CT, bone scan yielded a sensitivity, specificity, positive and negative predictive values of 87, 92, 99 and 49%, respectively. Hence, adding quantitative data analysis to bone scan interpretation can help to characterize more confidently malignant versus benign spinal and pelvic focal bone lesions, and thus increase the overall diagnostic performance of bone scintigraphy. The main limitations of this study remain the small subject population and lack of histological confirmation of the metastatic lesions.

## Data Availability

The datasets analyzed during the current study are available from the corresponding author on reasonable request.

## Abbreviations

95%CI: 95% confidence interval
^99m^Tc-DPD: ^99m^Tc-2,3-dicarboxy propane-1,1- diphosphonate
^99m^Tc-MDP: ^99m^Tc-methylene diphosphonate
NaF: Sodium fluoride
PSA: Prostate specific antigen
PSMA: Prostate specific membrane antigen
ROC: Receiver operating characteristics
SD: Standard deviation
SPECT/CT: Single-photon emission computed tomography coupled with computed tomography
SUV: Standardized uptake value
xSPECT: Quantitative xSPECT
